# A neurocomputational theory of how explicit learning bootstraps early procedural learning

**DOI:** 10.3389/fncom.2013.00177

**Published:** 2013-12-18

**Authors:** Erick J. Paul, F. Gregory Ashby

**Affiliations:** ^1^Beckman Institute for Advanced Science and Technology, University of Illinois at UrbanaChampaign, IL, USA; ^2^Department of Psychological and Brain Sciences, University of California, Santa BarbaraSanta Barbara, CA, USA

**Keywords:** basal ganglia, categorization, computational model, COVIS, declarative, non-declarative, procedural learning

## Abstract

It is widely accepted that human learning and memory is mediated by multiple memory systems that are each best suited to different requirements and demands. Within the domain of categorization, at least two systems are thought to facilitate learning: an explicit (declarative) system depending largely on the prefrontal cortex, and a procedural (non-declarative) system depending on the basal ganglia. Substantial evidence suggests that each system is optimally suited to learn particular categorization tasks. However, it remains unknown precisely how these systems interact to produce optimal learning and behavior. In order to investigate this issue, the present research evaluated the progression of learning through simulation of categorization tasks using COVIS, a well-known model of human category learning that includes both explicit and procedural learning systems. Specifically, the model's parameter space was thoroughly explored in procedurally learned categorization tasks across a variety of conditions and architectures to identify plausible interaction architectures. The simulation results support the hypothesis that one-way interaction between the systems occurs such that the explicit system “bootstraps” learning early on in the procedural system. Thus, the procedural system initially learns a suboptimal strategy employed by the explicit system and later refines its strategy. This bootstrapping could be from cortical-striatal projections that originate in premotor or motor regions of cortex, or possibly by the explicit system's control of motor responses through basal ganglia-mediated loops

## Introduction

The existence of multiple memory systems was theorized as early as the 1970s (Tulving, 1972), and the idea was more formally stated in the mid-1980s (Tulving, 1985). The distinction between declarative (i.e., explicit, conscious memory for specific events or facts) and non-declarative (i.e., implicit) memory systems is now well established (e.g., Poldrack and Packard, 2003).

The fundamental ideas driving multiple memory system theories have been applied more recently to the domain of category learning. Perhaps the most successful multiple system model of category learning is COVIS (Ashby et al., 1998). COVIS assumes two interacting systems that each map onto previously hypothesized human memory systems (Ashby and O'Brien, 2005). The COVIS explicit system uses declarative memory and mediates learning in tasks that require hypothesis testing, logical reasoning, and the application of verbalizable rules. In contrast, the procedural system uses non-declarative memory and learns to gradually associate motor programs with regions of perceptual space through reinforcement learning (Ashby and Waldron, 1999; Ashby et al., 2007).

Computational and mathematical models based on other theories of human category learning have been described, tested, and compared (e.g., Homa et al., 1979; Hintzman, 1984; Nosofsky, 1986; Kruschke, 1992; Ashby and Maddox, 1993; Smith and Minda, 2000; Love et al., 2004), and even other multiple-systems accounts have been proposed (Erickson and Kruschke, 1998; Anderson and Betz, 2001), though no other model has been formulated with such deep ties to known neurobiology as COVIS. It is precisely because of these neurobiological constraints that COVIS has been such a successful model of human category learning.

The neurobiology underlying its two distinct systems has been well described (Ashby et al., 1998; Ashby and Valentin, 2005; Ashby and Ennis, 2006). Furthermore, the neurobiological motivation of COVIS serves to constrain the model by utilizing the known neural basis of the constituent memory systems responsible for category learning to define the function and implementation of each system. Following is a brief description of the two systems of COVIS.

## Covis

As mentioned above, the explicit system of COVIS learns in tasks that require logical reasoning and explicit rules. This system tests simple hypotheses about category membership by allocating executive attention to single stimulus dimensions and then formulating explicit rules using Boolean algebra (e.g., logical conjunctions). Working memory is used to store candidate rules during testing. The COVIS explicit system is assumed to be mediated by a broad neural network that includes the prefrontal cortex (PFC), the anterior cingulate cortex (ACC), the head of the caudate nucleus, and medial temporal lobe (MTL) structures.

The procedural system of COVIS learns to associate motor programs with multidimensional stimuli via reinforcement learning (Ashby and Waldron, 1999). The neuroanatomical basis is the direct pathway through the basal ganglia. More specifically, for visual stimuli the relevant structures are early visual cortex (excluding V1) and parietal visual areas, posterior putamen, the internal segment of the globus pallidus (GPi), the ventral-anterior and ventral-lateral thalamic nuclei, and premotor cortex [i.e., supplementary motor area (SMA) and/or dorsal premotor cortex (PMd)]. The key site of learning is at cortical-striatal synapses, and plasticity there follows reinforcement learning rules, with dopamine from the substantia nigra pars compacta (SNpc) serving as the training signal.

**Figure 1:**
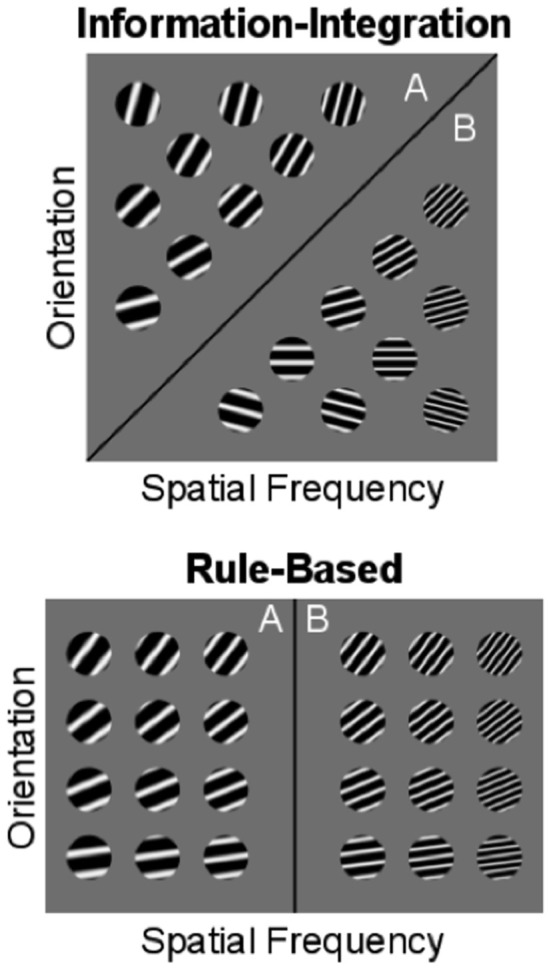
**Examples of information-integration and rule-based category-learning tasks**. The optimal boundary separating categories is shown as a solid black line.

COVIS assumes that the explicit system dominates in rule-based (RB) category-learning tasks. In RB tasks, the categories can be separated by a rule that is easy to describe verbally. In many cases, only a single stimulus dimension is relevant, although tasks where the optimal strategy is a logical conjunction are also RB. An example is shown in the bottom panel of Figure 1. Here, stimuli are discs with alternating black/white bars that vary in their orientation and thickness (or spatial frequency). A simple rule on the thickness dimension successfully accounts for the separation of the categories.

The top panel of Figure 1 shows an example of an information-integration (II) category–learning task. Here, information about both stimulus dimensions (i.e., orientation and spatial frequency) must be (pre-decisionally) integrated to respond optimally. Note that there is no way to verbalize the optimal strategy in this task (i.e., denoted by the diagonal boundary). RB strategies could be (and frequently are) adopted in II tasks, but such strategies lead to suboptimal performance. COVIS assumes that when the explicit system fails to learn a task of this kind, it passes control to the procedural system.

Many dissociations in learning RB and II categories have been observed across a variety of methodologies and in numerous human and non-human populations (see Ashby and Maddox, 2005, 2011 for reviews). These dissociations strongly support the hypothesis that multiple memory systems contribute to category learning.

## Interactions between declarative and procedural memory

Although overwhelming evidence now supports the existence of functionally separate declarative and procedural memory systems, much less is known about how these systems interact. The first important question to answer is how a single response is selected, given that either system can presumably control behavior?

Logically, there are at least three possible ways to select a single response when two learning and memory systems are simultaneously active. One possibility is that the outputs of the constituent systems are mixed or blended to produce the final output. This assumption is made by several currently popular categorization models (Erickson and Kruschke, 1998; Anderson and Betz, 2001). Mixture models assume that all categorization responses reflect a mixture of declarative and procedural processes, so that the difference between RB and II tasks is quantitative rather than qualitative—that is, the mixture might weight the declarative output more heavily in an RB task than in an II task, but some weight is always given to both systems. The problem for mixture models is to account for the many behavioral dissociations that have been reported between performance in RB and II tasks. For example, a simultaneous dual task greatly interferes with RB category learning, but not with II learning (Waldron and Ashby, 2001; Zeithamova and Maddox, 2006). If the dual task interferes with the use of declarative memory and responding is always a mixture of declarative and procedural memory processes then it seems that a dual task should interfere with both RB and II learning.

A second logical possibility, which we call *soft switching*, is that only one system controls each response, but that control is passed back and forth between the systems on a trial-by-trial basis. This is the assumption made by the original version of COVIS. More specifically, COVIS assumes that the response on each trial is generated by whichever system is most confident in the accuracy of its response (weighted by a system bias parameter).

**Figure 2:**
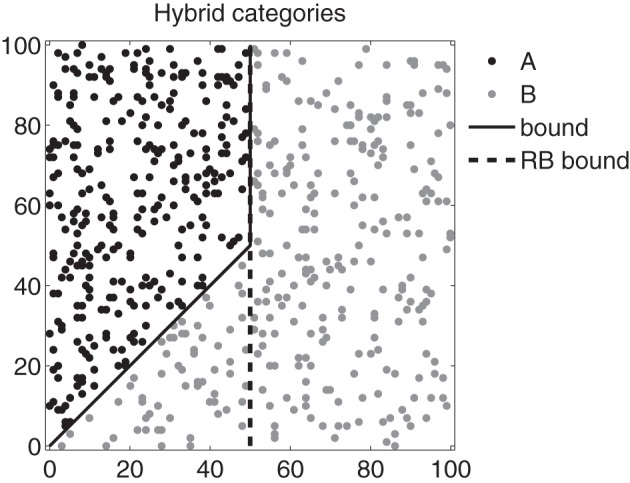
**Hybrid categories used by Ashby and Crossley (2010)**. Each black and gray dot marks the 2D coordinates of a stimulus (e.g., disks varying in spatial frequency and orientation as in Figure 1). The solid black line represents the optimal boundary; the dashed black line represents a suboptimal rule-based boundary. Note that the axes are in arbitrary units.

Ashby and Crossley (2010) reported results that argue against soft switching. These experiments used hybrid categories (illustrated in Figure 2) that were constructed so that perfect performance is possible if participants use a 1D rule on disks with steep orientations and an II strategy on disks with shallow orientations. Nevertheless, fewer than 5% of participants were able to adopt a strategy of the optimal type (solid black line). On the other hand, Erickson (2008) reported the results of an experiment in which about 40% of his participants appeared to switch successfully between declarative (i.e., RB) and procedural (i.e., II) strategies on a trial-by-trial basis. But this experiment provided participants with several explicit cues that signaled which memory system to use on each trial. Despite all of these cues, most participants failed to switch strategies. Thus, although the existing data suggest that some participants are able to soft switch under some conditions, the evidence argues strongly against soft switching as the default mechanism to resolve system interactions.

A third logical possibility is *hard switching* (HS), in which one system is used exclusively and a single switch is made to the other system (when the task demands it). This hypothesis seems most consistent with the available data—in that it accounts for all the RB vs. II behavioral dissociations, as well as the data of Erickson (2008) and Ashby and Crossley (2010). Nevertheless, hard switching faces numerous theoretical challenges. For example, consider an II category-learning task. A hard-switch version of COVIS assumes that participants begin experimenting with explicit strategies, and then when these all fail, switch to a procedural strategy. If so, then what is happening within the procedural system early in learning when declarative memory systems control behavior? One possibility is that no procedural learning occurs until the procedural system controls behavior. This model makes a strong prediction. A suboptimal explicit strategy will almost always yield performance that is significantly above chance in an II task. Thus, if the procedural system cannot learn while the explicit system controls behavior, then a hard-switch model must predict that accuracy will rise to well above chance and then suddenly fall back to chance when control is passed to the procedural system. To our knowledge, none of the many published II studies has ever reported this strange data pattern.

The second possibility is that the procedural system learns on trials when the explicit system is controlling behavior. COVIS actually predicts such learning because it assumes that each system receives its own separate feedback signal that rewards or punishes the system for recommending either the correct or incorrect response, respectively, regardless of whether that recommendation was used to select the single response made on that trial. These separate feedback signals allow both systems to learn independently of the other, and regardless of whether or not they control responding.

The assumption that the explicit and procedural category-learning systems each receive their own independent feedback signal on every trial is very strong. In most category learning experiments, only a single feedback signal is given that is based entirely on the emitted (observable) response; independent feedback would require sophisticated self-monitoring that might for example, send a reward signal to the procedural system on trials when the emitted response was incorrect. The explicit system might have such flexibility: evidence suggests that the PFC can handle abstract forms of feedback and generate second-order feedback (Ashby et al., 2002; Maddox et al., 2003; Cools et al., 2006; Maddox et al., 2008; Wallis and Kennerley, 2010). On the other hand, the available evidence does not support the hypothesis that the procedural system can self-monitor and generate its own feedback. Learning in the procedural system depends on dopamine signals in the striatum. Dopamine neurons in SNpc respond to rewards and reward-predicting stimuli, and also encode reward prediction error (Schultz et al., 1997, 2000; Tobler et al., 2003), which depends in part on the valence of the feedback. This suggests that the basal ganglia are unable to flexibly manipulate feedback signals, and instead respond to the valence (and expectation) of feedback (and reward). As such, the possibility of independent feedback seems unlikely.

A more plausible solution is to assume that each system receives the feedback elicited by the observable response and that each system must use this common feedback to guide learning. The theoretical challenge for this hypothesis is to show how one system can learn from feedback that is based on the response of the other system. Because the evidence is good that the explicit system dominates during early responding, this question is moot for any tasks that are learned by the explicit system (e.g., RB tasks), because in such cases, evidence suggests that the procedural system is never used. In II tasks however, we expect the explicit system to control early learning and the procedural system to control late learning. The key question addressed by this article is how the procedural system can learn during trials when the explicit system controls responding and there is only a single source of feedback.

To begin, we need to identify a candidate neural mechanism that mediates the competition between the two systems. The hypothesis that the procedural system is able to learn while the explicit system is in control suggests that when in control, the explicit system prevents the procedural system from accessing motor output systems, but does not interfere with learning. Some independent evidence supports this hypothesis (e.g., Packard and McGaugh, 1996; Foerde et al., 2006, 2007).

Given these considerations, Ashby and Crossley (2010) proposed that frontal cortex and the subthalamic nucleus (STN) control system interactions via the hyperdirect pathway through the basal ganglia. The hyperdirect pathway begins with direct excitatory glutamate projections from frontal cortex (via presupplementary cortex, preSMA) to the STN, which sends excitatory glutamate projections directly to the internal segment of the globus pallidus (GPi; Parent and Hazrati, 1995; Joel and Weiner, 1997), making it more difficult for striatal activity to affect cortex. The evidence that the preSMA is in this circuit comes from several sources. First, Hikosaka and Isoda (2010) reviewed evidence that the preSMA is crucial for switching between controlled and automatic responding. Second, Waldschmidt and Ashby (2011) reported that after 20 sessions of practice, the only brain areas in which neural activation correlated with accuracy in an II task were the preSMA and SMA.

Evidence for a role of the STN in this model comes largely from studies using the stop-signal task where participants initiate a motor response as quickly as possible when a cue is presented. On some trials, a second cue is presented soon after the first signaling participants to inhibit their response. A variety of evidence implicates the STN in this task (Aron and Poldrack, 2006; Aron et al., 2007; Mostofsky and Simmonds, 2008). A popular model is that the second cue generates a “stop signal” in cortex that is rapidly transmitted through the hyperdirect pathway to the GPi, where it cancels out the “go signal” being sent through the striatum.

Ashby and Crossley (2010) hypothesized that when the explicit system is controlling behavior a stop signal may inhibit a potentially competing response generated by the procedural system: the PFC could increase STN output via the hyperdirect pathway, preventing the procedural system from influencing cortical motor systems, thereby allowing the explicit system to control the overall response. Note that because the inhibition occurs downstream from the striatum, this hypothesis theoretically allows procedural learning to occur within the striatum. The next section describes several different model architectures that will be used to explore the conditions under which procedural learning can occur with a single source of feedback.

## Simulation set 1—COVIS architectures

The basic model that we will use in all investigations is COVIS (Ashby et al., 1998, 2011). The original version of COVIS assumed soft switching and independent feedback. However, to gain more insight into system interactions, we will explore a number of alternative model architectures. For the reasons described above, our focus will be on two model features: the nature of the feedback signal (separate signals to each system vs. a single feedback signal); and the switching mechanism (soft vs. hard). Following are descriptions of three alternative versions of COVIS that explore the effects of varying these two features.

### Model 0: independent feedback, soft switching

Our first goal is to implement COVIS computationally as described in Ashby et al. (2011). Only components critical to the simulations will be described; interested readers are encouraged to review the complete description elsewhere (e.g., Ashby et al., 1998, 2011; Hélie et al., 2012a,b). Model 0 is simply the independent feedback model previously described with some simplifications made to each system to improve the likelihood of learning in the procedural system, and to maximize computational efficiency.

First, because our goal is not to elucidate the nature of learning in the explicit system, it was heavily simplified to respond with the most accurate possible one-dimensional rule from the outset of the simulated experiment (in normal applications, the explicit system selects and tests a variety of rules). Thus, in current applications, the explicit system has no free parameters and reduces to the equation below. The response rule is: respond A on trial *n* if *h*_*E*_(*x*)< 0; respond B if *h*_*E*_(*x*)> 0; and the discriminant function is defined as:

(1) $$h_{E}\left(x\right)=x_{d}-C_{d}-\varepsilon_{E},$$

where *x*_*d*_ is the value of stimulus *x* on dimension *d*, *C*_*d*_ is a constant that plays the role of a decision criterion (typically learned; hard-coded here) and ε _*E*_ is a normally distributed random variable with mean 0 and variance σ^2^_*E*_ that models the variability in both stimulus perception and memory of the decision criterion. When σ^2^_*E*_ is large, the discriminant value becomes less deterministic, so σ^2^_*E*_ was set to zero in the present application.

The COVIS procedural system is a two-layer connectionist network. The input layer includes 625 input units arranged in a 25 × 25 grid (i.e., because the simulations will use stimuli that vary on two stimulus dimensions). Each input unit is tuned to a particular stimulus, in the sense that it is maximally activated when its preferred stimulus is presented, and it is less activated when similar stimuli are presented. The activation in sensory cortical unit *K* on trial *n* is given by

(2) $$I_{K}(n)=e^{\frac{-d{\left(K,\text{ stimulus}\right)}^{2}}{\sigma_{R}}}$$

where *d*(*K*, stimulus) is the Euclidean distance (in stimulus space) between the stimulus preferred by unit *K* and the presented stimulus (i.e., in units of 25× 25 space). Equation 2 is a radial basis function (e.g., Kruschke, 1992; Riesenhuber and Poggio, 1999). The constant σ_*R*_ has the effect of expanding or narrowing the width of the radial basis function, much like a variance.

The output layer in the COVIS procedural system is assumed to represent the striatum. The model includes the same number of output units as there are alternative responses. All simulations described here used two categories (A and B), so all models included two output units. Activation of striatal unit *J* in the output layer is determined by the weighted sum of activations from the input layer projecting to it:

(3) $$S_{J}(n)=\sum_{K=1}^{625}w_{K,J}(n)I_{K}(n)$$

where *w*_*K, J*_ (*n*) is the strength of the synapse between cortical unit *K* and striatal cell *J* on trial *n*. The decision rule in the procedural system is similar to that of the explicit system. The decision rule is: respond A on trial *n* if *S_A_*(*n*) > *S_B_*(*n*); otherwise respond B. The relative activity between striatal units changes as the model learns, and learning is accomplished by adjusting the synaptic weights, *w*_*K, J*_(*n*), up or down from trial-to-trial via reinforcement learning.

The weights in the procedural system are updated based on the three factors: (1) pre-synaptic activation, (2) post-synaptic activation, and (3) dopamine levels (e.g., Arbuthnott et al., 2000; Ashby and Hélie, 2011). Specifically, *w*_*K, J*_(*n*) is updated after each trial using the following learning rule:

(4) $$\begin{array}{l} w_{K,J}(n+1)\text{​}=\text{​}w_{K,J}(n)+\alpha I_{K}(n){\left[S_{J}(n)-\theta_{\text{NMDA}}\right]}^{+}\\ \;{\left[D(n)-D_{\text{base}}\right]}^{+}\left[w_{\text{max}}-w_{K,J}(n)\right]\\ \;-\beta I_{K}(n){\left[S_{J}(n)-\theta_{\text{NMDA}}\right]}^{+}{\left[D_{\text{base}}-D(n)\right]}^{+}w_{K,J}(n)\\ \;-\gamma I_{K}(n){\left[\theta_{\text{NMDA}}-S_{J}(n)\right]}^{+}{\left[S_{J}(n)-\theta_{\text{AMPA}}\right]}^{+}w_{K,J}(n) \end{array}$$

The function [*g*(*n*)]^+^ = *g*(*n*) if *g*(*n*) > 0, and otherwise *g(n)* = 0. The constant *D*_base_ is the baseline dopamine level, *D*(*n*) is the amount of dopamine released on trial *n* following feedback, and α, β, γ, θ_NMDA_, and θ_AMPA_ are all constants. The first three of these (i.e., α, β, and γ) operate like standard learning rates because they determine the magnitudes of increases and decreases in synaptic strength. The constants θ_NMDA_ and θ_AMPA_ represent the activation thresholds for post-synaptic NMDA and AMPA (more precisely, non-NMDA) glutamate receptors, respectively. The numerical value of θ_NMDA_ >θ_AMPA_ because NMDA receptors have a higher threshold for activation than AMPA receptors. This is critical because NMDA receptor activation is required to strengthen cortical-striatal synapses (Calabresi et al., 1996).

The positive term in Equation (4) describes the conditions under which synapses are strengthened (i.e., striatal activation above the threshold for NMDA receptor activation and dopamine above baseline) and the two negative terms describe conditions that cause the synapse to be weakened. The first possibility (line 3) is that post-synaptic activation is above the NMDA threshold but dopamine is below baseline (as on an error trial), and the second possibility is that striatal activation is between the AMPA and NMDA thresholds. Note that synaptic strength does not change if post-synaptic activation is below the AMPA threshold.

The Equation (4) model of reinforcement learning requires that the amount of dopamine released in response to the feedback signal [that is, the *D*(*n*) term] is specified on every trial. COVIS adopts the popular model that, over a wide range, dopamine firing is proportional to the reward prediction error (RPE) (e.g., Schultz et al., 1997; Tobler et al., 2003):

(5) RPE=Obtained Reward−Predicted Reward

The procedural system of COVIS uses a simple model of dopamine release by first computing both obtained and predicted reward, and then by estimating the amount of dopamine released as a function of RPE.

In applications that do not vary the valence of the rewards, the obtained reward *R_n_* on trial *n* is defined as

(6) $$R_{n}=\left\{ \begin{array}{l} +1\text{ if correct feedback}\\ \;0\text{ if no feedback is given}\\ -1\text{ if error feedback} \end{array}\right.$$

In models that assume separate, independent feedback signals, the reward signal is based on the response suggested by the procedural system regardless of which system initiated the response. In models that assume a single feedback signal, the reward is based on the observable response. The single-operator learning model (Bush and Mosteller, 1955) is used to compute predicted reward, *P_n_*:

(7) $$P_{n}=P_{n-1}+\alpha_{\text{pr}}\left(R_{n-1}-P_{n-1}\right)$$

It is well known that when computed in this fashion, *P_n_* converges exponentially to the mean reward value and then fluctuates around this value (until reward contingencies change). The parameter α_pr_ governs how quickly *P_n_* converges.

Finally, to compute the dopamine release to the feedback, a simple model matching empirical results reported by Bayer and Glimcher (2005) is used:

(8) $$D(n)=\{\begin{array}{cc} 1 & \text{if RPE}>1\\ 0.8\text{RPE}+0.2 & \text{if }-0.25\;\leq \text{RPE}\leq 1\\ 0 & \text{if RPE}<-0.25 \end{array}$$

Note that the baseline dopamine level is 0.2 (i.e., when RPE = 0) and that dopamine levels increase linearly with RPE between a floor of 0 and a ceiling of 1.

For the final simplification, a strong form of lateral inhibition at the level of the striatum was assumed. Computationally, this amounts to updating only the weights associated with the striatal unit matching the response suggested by the procedural system. For example, if the procedural system suggests an “A” response, only the weights associated with the “A” striatal unit are modified. This simplification effectively serves a dual-purpose: it accelerates learning in the procedural system because only the weights relevant to that trial are updated and improves computational efficiency.

In order to resolve the competition between the systems and to select an overall model response, confidence is measured on every trial by calculating a discriminant value for each system. For the explicit system, the discriminant value equals the distance from the stimulus to the decision bound used by the explicit system. The confidence of the explicit system, which equals |*h_*E*_*(*n*)|, will be large on any trial where the stimulus is far (in stimulus space) from the response criterion. In the procedural system, confidence is measured by the difference in striatal unit activity and is defined by:

(9) $$\left|h_{P}(n)|=|S_{A}(n)-S_{B}(n)\right|$$

Note that as with the explicit system, the procedural system will be highly confident when it strongly favors one response over another. Hence, the explicit system is more confident when the stimulus is far from the bound and the procedural is more confident when the stimulus is strongly associated with one motor response but not the other.

The trust placed in each system is determined by overall system weights, θ_*E*_(*n*) and θ_*P*_(*n*), where θ_*E*_(*n*) + θ_*P*_(*n*) = 1. Because humans are naturally rule preferring and there is no procedural learning at the beginning of an experiment, COVIS assumes that trust in the explicit system is initially much higher than in the procedural system, hence θ_*E*_(1) = 0.99. Throughout each experiment, the system weights are adjusted based on the success of the explicit system. When the explicit system suggests a correct response,

(10) $$\theta_{E}(n+1)=\theta_{E}(n)+\Delta_{\text{OC}}\left[1-\theta_{E}(n)\right],$$

where Δ_OC_ is a learning rate constant. If instead the explicit system suggests an incorrect response then

(11) $$\theta_{E}(n+1)=\theta_{E}(n)-\Delta_{\text{OE}}\theta_{E}(n),$$

where Δ_OE_ is another rate constant. The two regulatory terms on the ends of Eqs. 10 and 11 restrict θ_*E*_(*n*) to the range 0 ≤ θ_*E*_(*n*) ≤ 1. Finally, on every trial, θ_*P*_(*n*) = 1 − θ_*E*_(*n*). Thus, Equations 10 and 11 also guarantee that θ_*P*_(*n*) falls in the range 0 < θ_*P*_(*n*) < 1. The parameters Δ_OC_ and Δ_OE_ control how fast θ_*E*_(*n*) changes in response to correct and incorrect feedback, respectively; thus, they also control how quickly θ_*P*_(*n*) changes, which is related to how frequently the procedural system will be allowed to generate the overall system response.

The overall system decision rule is to emit the response suggested by the explicit system if θ_*E*_(*n*) × | *h*_*E*_(*n*)| > θ_*P*_(*n*) × |*h*_*P*_(*n*)|; otherwise emit the response suggested by the procedural system. Notice that this is done on a trial-by-trial basis and that either system may be responsible for the overall response generated depending on the confidence and trust; hence, this a soft-switching model.

All exploratory analyses will be conducted with this model, so that Model 0 will serve as a baseline to compare the effects of modifications to this standard implementation. The simplifications made to the model serve only to optimize learning performance and computational efficiency. It is important to emphasize that the simplifications will only help the procedural system learn faster, so all results should be interpreted as a best-case learning scenario.

### Model 1: single source of feedback, hard switch

The first modified COVIS model follows the revisions suggested by the evidence reviewed above. Specifically, this model only receives a single source of feedback based on the response of the controlling system and assumes that the switch from the explicit to the procedural system is a one-time, hard switch.

The goal of the present research is not to specify exactly how this hard switch is implemented computationally, but instead to evaluate learning in the procedural system under explicit-system controlled responding. For this reason, Model 1 never switches from the explicit to the procedural system. The hyperdirect pathway model places the switching gate downstream from learning in the procedural system (i.e., downstream from the striatum), so the procedural system weights are still modified on every trial. The absence of any switching is a worst-case scenario for a hard-switch model in a procedurally learned task, but it is also the best way to evaluate the ability of the procedural system to learn while the explicit system controls responding (by design, the procedural system will necessarily learn after a switch).

With a single feedback source, and because the model never switches, RPE (Equations 5 and 7) and therefore dopamine Equation (8) will always be driven by the responses of the explicit system. Concretely, reward, *R_n_* is determined only by the explicit system's response to the stimulus (affecting RPE and dopamine calculations). The procedural system will suggest its response normally, but the weights will be updated based on the feedback elicited by the explicit system response. All other features of this model are identical to the implementation of Model 0.

### Model 2: single source of feedback, soft switch

The second modified COVIS model is a simple modification of Model 0 in which a single source of feedback is used in conjunction with soft switching. Models 1 and 2 together allow procedural-system learning with a single source of feedback to be evaluated both under hard- and soft-switching architectures.

As with Model 0, the switching algorithm described above was used to select between procedural and explicit system responses on every trial. The difference from Model 0 is that, while the explicit system controls the overall response, the procedural system (specifically, RPE and dopamine) is updated using the feedback signal generated by the explicit system's response. On the other hand, whenever the procedural system controls the overall response, the feedback signal is guaranteed to be congruent with the response suggested by the procedural system. All other features of this model are identical to the implementation of Model 0.

A summary of these models and their differences appears in Table 1.

**Table 1:** **Summary of each model and brief description of differences**.

**Name**	**Feedback type**	**Switching type**	**Description**
Model 0 (2FB-SS)	Independent (2FB)	Soft (SS)	Original formulation of COVIS where each system receives independent feedback and either system controls responding on a trial-by-trial basis.
Model 1 (1FB-HS)	Single (1FB)	Hard (HS)	Each system is only updated with a single feedback signal from the responding system; switching from explicit to procedural responding occurs once.
Model 2 (1FB-SS)	Single (1FB)	Soft (SS)	Each system is only updated with a single feedback signal from the responding system; system switching is on a trial-by-trial basis.

## Method—simulation set 1

### Categorization simulations

#### Information-integration categories

**Figure 3:**
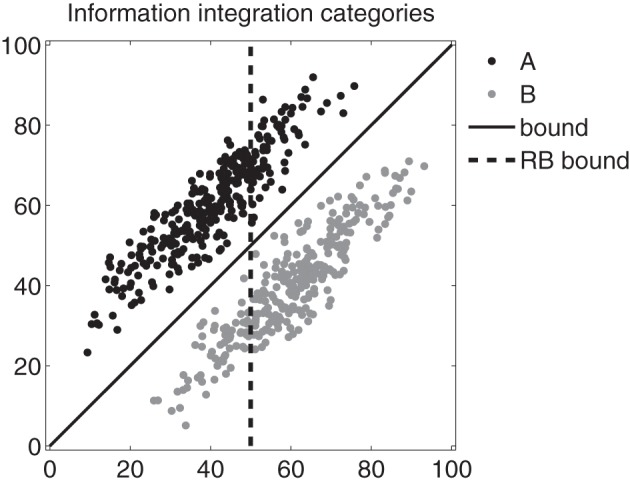
**Information integration (II) categories used in the simulation experiments**. Each black and gray dot marks the 2D coordinates of a stimulus (e.g., disks varying in spatial frequency and orientation as in Figure 1). The solid black line represents the optimal boundary; the dashed black line represents a suboptimal rule-based boundary. Note that the axes are in arbitrary units.

Each model was evaluated through simulation to determine whether the procedural system could learn II categories (e.g., as in Figure 1, top panel). Human participants reliably learn categories like these, so any multiple systems model of category learning must pass this basic performance benchmark. These simulations served as a first-pass elimination round—any model performing poorly on this category structure should be considered unviable while all successful models will move on to a second simulation experiment. A total of 300 sample stimuli were drawn from each of two bivariate normal distributions (with the category means and variances in Table 2). The sample category distributions used are shown in Figure 3. Maximum performance by the explicit system on these categories is 78.5%, and the explicit one-dimensional bound (the dashed line) was set to guarantee that the explicit system reached this level of performance.

**Table 2:** **Mean, variance, and covariance parameters for each category in Figure 3**.

**Category**	**μ_*x*_**	**μ _*y*_**	**σ^2^_*x*_, σ^2^_*y*_**	***Cov*_*x, y*_**
A	40	60	167.59	151.26
B	60	40	167.59	151.26

Model 0 should have no trouble learning these categories as each subsystem receives independent feedback. Models 1 and 2 are particularly interesting because, currently, it is unknown if any COVIS model will successfully learn II categories without independent feedback.

#### Hybrid categories (Ashby and Crossley, 2010)

All models that successfully learned the benchmark II category structures were tested on the hybrid categories shown in Figure 2 (Ashby and Crossley, 2010). These categories require a 1D rule on disks with steep orientations and an II strategy on disks with shallow orientations, so optimal responding requires trial-by-trial system switching. As mentioned earlier, Ashby and Crossley (2010) reported that only 4% of participants showed any evidence of trial-by-trial switching. An obvious prediction is that soft-switching models will perform well on these categories (i.e., better than human participants), but it is not clear how well hard-switching models will perform.

The hybrid categories shown in Figure 2 were used in this simulation. A total of 300 stimuli were uniformly sampled from each of two categories separated by the hybrid bound. Maximum performance by the explicit system on these categories is 88.33%, and the one-dimensional bound of the explicit system (the dashed line) was set to this optimal position.

### Assessment of model performance—parameter space partitioning

The goal of our simulation analyses is not to ask how well each particular model can fit some data set, but rather to ask whether each model is or is not capable of learning. Before concluding that a model cannot learn, it is vital to examine its performance under a wide range of parameter settings. Similarly, when a model does learn, it is important to know whether the learning is representative of the model, or restricted to a small set of parameter settings. Because of these unique modeling goals, we chose to evaluate the performance of each model using a parameter space partitioning (PSP) analysis (Pitt et al., 2006, 2008). PSP is a technique used to investigate the global performance of cognitive models. The basic idea is to exhaustively explore the parameter space (defined by the free parameters of the model) and to map out regions that lead to qualitatively different behaviors (called data patterns).

The end result is a disjoint partitioning of the parameter space into regions that each produces a qualitatively unique behavior (data pattern). The finite set of data patterns must be defined by the experimenter. In order to map out the space, discrete “steps” in the parameter space are each assigned a data pattern. A step is defined as a particular set of numerical values for every parameter that defines the space. Because the computational demands of searching the parameter space increase dramatically with the number of parameters, PSP uses an efficient Markov chain Monte Carlo search algorithm (Pitt et al., 2006). Recall that the implementation of COVIS used here was optimized for computational efficiency so that the parameter space search would be computationally tractable. Once the entire parameter space has been mapped, volumes (i.e., contiguous regions) of the space are computed to quantify the range of parameters that produce a particular data pattern. These volumes describe how likely a behavior is to be produced by the model.

**Figure 4:**
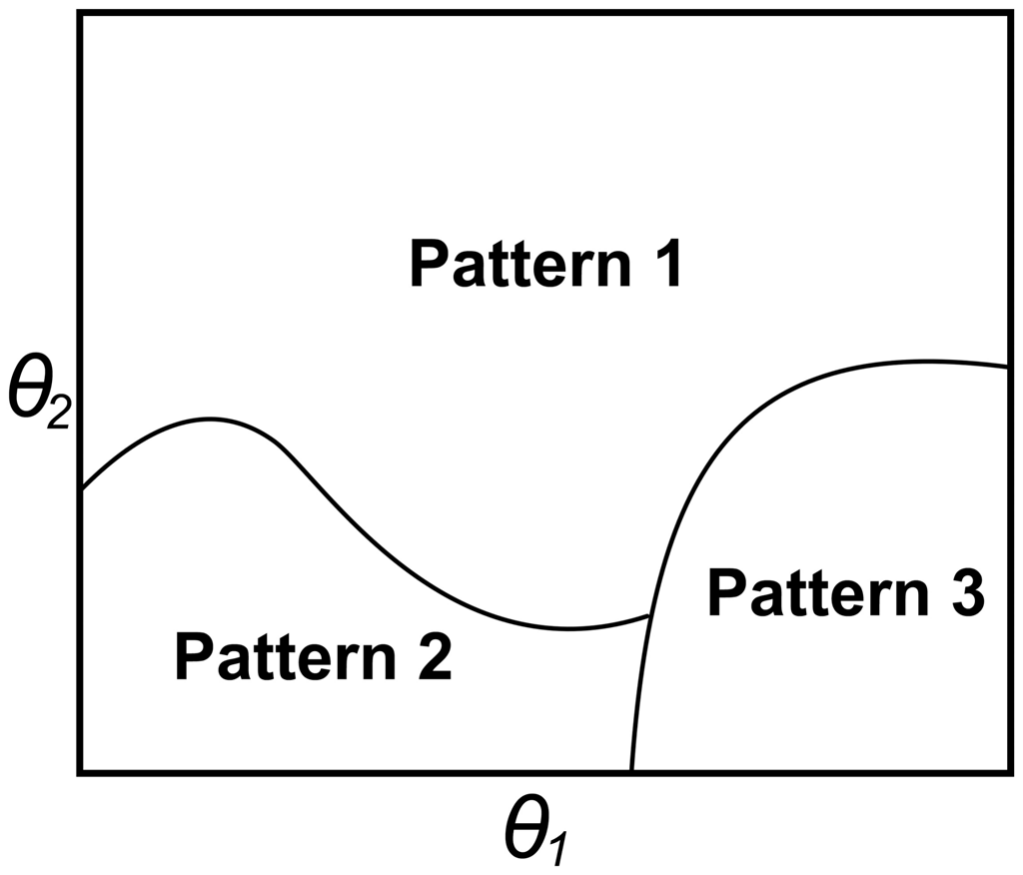
**A hypothetical example of parameter space partitioning (PSP) for a model with two parameters, (θ_1_ and θ_2_)**. In this example, varying these two parameters allows the model to produce three qualitatively different data patterns. The PSP algorithm efficiently steps through the space to determine which data pattern is produced by each combination of parameters. The curves separate regions where different patterns are predicted. The PSP algorithm returns the area (or volume) of each of these three regions. In this example, pattern 1 accounts for more than 50% of the total area.

Figure 4 offers a schematic representation of this analysis and related concepts. In this hypothetical example, a simple model is defined by two parameters (θ_1_, θ_2_). By simultaneously varying these parameters, the model can account for three qualitatively different behaviors or data patterns. These could be almost anything. For example, pattern 1 might be that performance is significantly better in an experimental condition than in a control condition (e.g., at least 5% better), pattern 2 could be the reverse ordering, and pattern 3 might be that performance in the two conditions is not significantly different. The PSP analysis measures the area (or volume when there are 3 or more parameters) of the parameter space that predicts each of the three patterns. In this case, the analysis reveals that for most pairs of parameters, data pattern 1 is produced, but for more restricted sets of parameters, patterns 2 or 3 occur.

### Implementation of PSP

In the following analyses, a MATLAB implementation of the PSP algorithm was obtained from the website of J. I. Myung (http://faculty.psy.ohio-state.edu/myung/personal/psp.html). For the PSP search algorithm to proceed, the model must produce deterministic output (i.e., produce the same behavior) for a given set of parameters. In order to accomplish this, all randomized features of the model must be fixed. All models used here omit the noise (e.g., perceptual and criterial) terms typically included. Thus, the only probabilistic features are the random initial weights for each striatal unit and the randomized stimulus ordering.

In normal applications of the model the stimulus ordering is completely randomized in every simulation. Because of this, the performance of the model averaged across many (i.e., 100 or more) simulations will be robust to stimulus ordering. This is important because Pitt et al. (2006) observed that other models of category learning (e.g., ALCOVE; Kruschke, 1992) are sensitive to stimulus ordering. In addition, the effect of stimulus ordering interacts with the particular initial randomization of weights in the procedural system. To handle these random ordering and initialization effects, it is necessary to choose a fixed random sample on which to run all PSP analyses. For these reasons a random set of 19 random stimulus orderings and weight initializations were generated. Each simulation (each step in the parameter space) was tested on this set, and the model performance was averaged across them. This allowed for deterministic output and relatively stable estimates of the model behavior.

It is necessary to select the parameters defining the parameter space. Including every parameter of the model would be inefficient because the parameter space would be very high dimensional and many parameters interact in predictable ways. For example, in COVIS, if the AMPA and NMDA thresholds (Equation 4) are set too high, then the procedural system will be unable to learn regardless of the values of any other parameters.

Our goal is only to evaluate the ability of learning to proceed in the procedural system, so the parameter space was constrained to parameters that directly affect procedural learning: the learning rates, α and β (Equation 4), and α_pr_ (Equation 7), which determines how fast predicted reward converges to the expected reward value, and therefore affects the trial-by-trial dopamine fluctuations and weight adjustment.

Additionally, for Model 2 only, the system switching rate parameters Δ_OC_ and Δ_OE_ (Equations 10, 11) were also included in the PSP analysis. These parameters directly affect system-switching behavior and learning in the procedural system (note that this is not true for model 0 because the procedural system always receives independent feedback). Table 3 summarizes the function and search range for each manipulated parameter. Every other parameter was fixed to the specific value shown in Table 4. The learning rate parameter γ (Equation 4) was irrelevant because the AMPA and NMDA thresholds were set low enough that the model would never be above AMPA but below NMDA (e.g., line 3 of Equation 4).

**Table 3:** **Function and search range for each parameter in the PSP analysis**.

**Parameter**	**Function**	**Range**
α_pr_	Controls how fast predicted reward converges, affecting dopamine.	[0.005, 1]
α	Learning rate for strengthening weights.	[0.001, 1]
β	Learning rate for weakening weights.	[0.001, 1]
Δ_OC_	Controls how explicit system bias grows for correct responses	[0.001, 0.2]
Δ_OE_	Controls how explicit system bias decays for incorrect responses	[0.001, 0.2]

**Table 4:** **Values of all parameters that remained fixed across all simulations**.

**Parameter**	**Function**	**Value (for each model)**
σ_R_	Width of the RBF	4.5
θ_AMPA_	AMPA threshold	0.01
θ_NMDA_	NMDA threshold	0.1

The next choice in a PSP analysis is to define the qualitative behaviors (data patterns) that determine the partitioning of the parameter space. We chose to partition based on the accuracy of the procedural system separated into deciles from 0 to 100%; hence, a total of 10 data patterns were possible. For example, data pattern 1 occurs if the procedural system accuracy falls between 0 and 10%, pattern 2 occurs if accuracy falls between 10 and 20%, and so forth. This simple definition of data patterns easily allows for a rough quantification of how well the procedural system performs on the category structure after the weights have been trained.

The PSP analysis proceeded in two stages: the initial partitioning stage, and an evaluation of robustness stage. For the initial stage, each step in the parameter space was tested on all 19 initializations. Each initialization included a training phase, where the model was trained with feedback on the sample stimuli, and a testing phase where end-of-simulation (learned) procedural system weights were run forward through the stimuli again simply to estimate the asymptotic procedural system accuracy. The test performance of the model was averaged over all 19 initializations to determine the final data pattern for each step in the parameter space. The PSP algorithm proceeded for six search cycles to obtain a reliable partitioning of the parameter space.

The complete PSP search returned the volume of parameter space that was associated with each of the 10 data patterns, and a specific set of parameter values that generated each discovered pattern. The robustness stage further tested each model in 200 random stimulus orderings and weight initializations using the parameters returned for each discovered data pattern. This two-stage approach was used because the initial PSP analysis is computationally demanding, so running hundreds of simulations per step in parameter space would be prohibitively time consuming. The second stage is important, though, because it essentially establishes the reliability of the parameters to produce a particular behavior (pattern).

## Results—simulation set 1

### Model 0: independent feedback, soft switch

Recall that Model 0 is simply the COVIS model as previously described. It is an independent feedback model (i.e., both systems learn independently and receive a separate feedback signal), and uses soft switching (either system can potentially control the overall system response on every trial). This version of Model 0 will be referred to as Model 0 (2FB-SS) because it receives two independent feedback (2FB) signals and uses soft switching (SS).

#### Information integration categories

**Figure 5:**
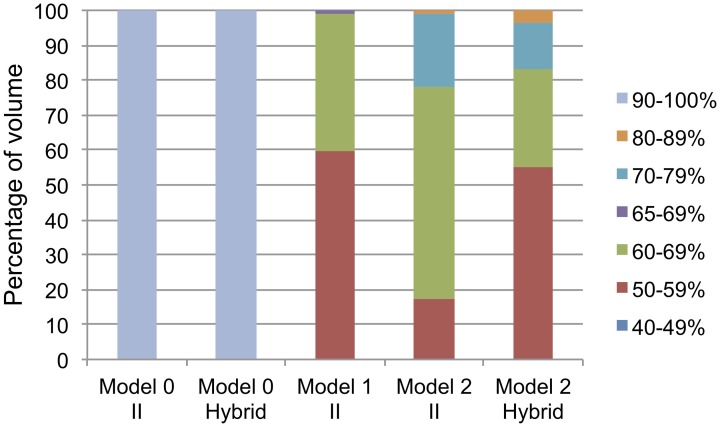
**Percentage of volume of the parameter space for each data pattern discovered for every model and category structure**. The legend corresponds to test phase accuracy of the procedural system (i.e., possible data patterns). Each color represents a unique data pattern discovered by the PSP for that combination of model/category structure, and the height of the color in the bar corresponds to the volume of parameter space producing that data pattern. The range from 65 to 69% is a special pattern only searched for in model 1 (see text for details).

Figure 5 shows the percentage of the volume of parameter space that produced each data pattern that was discovered. Note that Model 0 (2FB-SS) showed robust learning of the II categories in the majority of the parameter space (Figure 5, first column). A total of three data patterns were found. Over 99.99% of the volume of the parameter space learned the categories at or above 90% accuracy (Pattern 10). The remaining two data patterns accounted for less than 0.01% of the parameter space volume and corresponded to accuracy in the 70–79% and the 80–89% deciles (Patterns 8 and 9).

Recall that in the Stage 2 analysis, representative parameter values were chosen that produced each of the three observed data patterns and then the model was tested on 200 random stimulus orderings and weight initializations for each of these three parameter settings. In all cases, the performance of the model on these new tests was virtually identical to the performance on the 19 stimulus orderings and weight initializations used in the PSP analysis. Thus, learning in Model 0 (2FB-SS) is highly robust.

**Figure 6:**
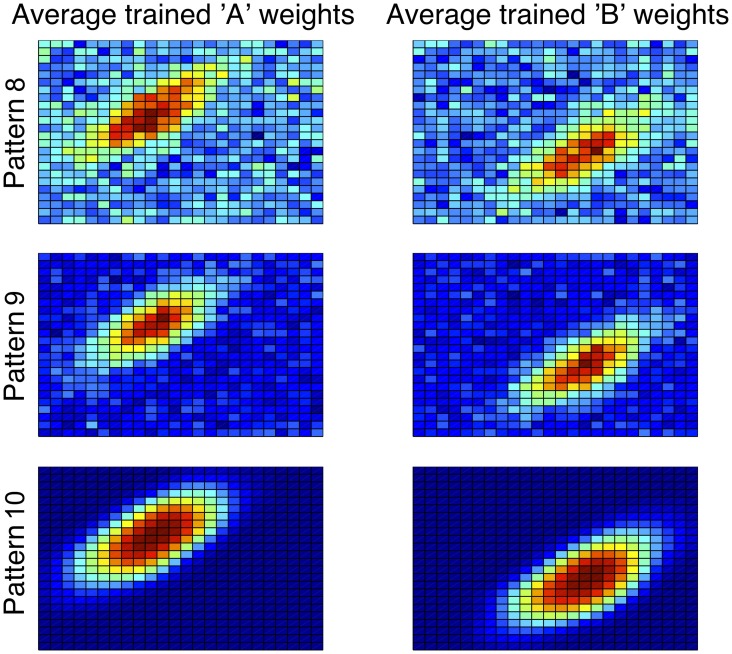
**“A” and “B” striatal weights in the procedural system of model 0 (2FB-SS) after training in information integration categories, averaged across 200 simulations**. Blue (cool colors) represent small weights whereas red (warm colors) represent large weights. Patterns 8, 9, and 10 refer to the 70–79, 80–89, and 90–100% performance deciles, respectively. Note the good correspondence between the high-performing bottom row weights (Pattern 3) and the stimuli plotted in Figure 3, and also the weaker correspondence in the lower-performing weights.

Finally, Figure 6 shows the learned procedural system weights for each striatal unit at the end of training (again, averaged across 200 simulations). It is clear from the figure that a progression of weight strength follows the data patterns: parameters producing the worst model performance led to noisier, smaller weights than parameters producing the best model performance. Still, the success of Model 0 (2FB-SS) in the II categories suggests that it will learn equally well in the hybrid category structures.

#### Hybrid categories

As with the II categories, Model 0 (2FB-SS) showed very good learning throughout the parameter space with the hybrid categories (Figure 5, second column). A total of five data patterns were found. Over 99.94% of the parameter space learned the hybrid categories at or above 90% accuracy. Less than 0.06% of the parameter space was accounted for by data patterns corresponding to the four accuracy deciles between 50 and 90%.

As with the II categories, Model 0 (2FB-SS) showed very robust learning in each region of the parameter space. In all cases, performance on the 200 random initializations was virtually identical to performance on the 19 training initializations. The behavior of the model appears to be very similar for both hybrid and II categories. An examination of the weights showed that they closely mimicked the structure of the hybrid categories (e.g., as the Figure 6 weights mimic the structure of the II categories).

### Model 1: single source of feedback, hard switch

Recall that Model 1 receives only one feedback signal, and uses hard switching (the explicit system controls responding until it yields control to the procedural system, at which point the procedural system controls responding), so we refer to this model as Model 1 (1FB-HS). Also recall that in these simulations the procedural system is never allowed to respond—in other words, the hard switch never occurs. This way, the ability of the procedural system to learn during explicit system responding can be fully evaluated.

#### Information integration categories

The partitioning for Model 1 (1FB-HS) required a slight modification to acquire a higher resolution partitioning of the space. In the first partitioning, approximately 62% of the parameter space volume was between 40 and 59% test performance accuracy, and about 38% of the volume was associated with test performance accuracy between 60 and 69%. To create a finer-grained partitioning, for Model 1 (1FB-HS) only the 60–69% decile was subdivided into 60–64 and 65–69% ranges.

**Figure 7:**
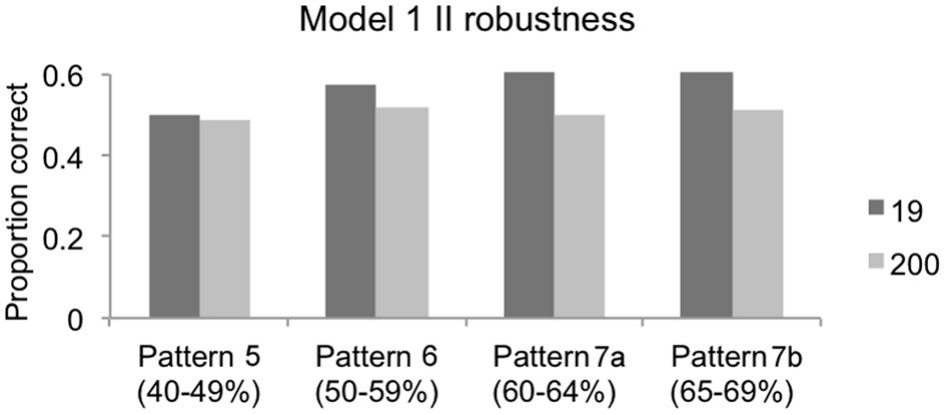
**Robustness of Model 1 (1FB-HS) in II categories for each of four discovered data patterns**. Dark gray bars represent the performance of the model (proportion correct in the test phase) for the 19 fixed initializations used in the PSP analysis; light gray bars represent the performance of the model across 200 random initializations. In every case, the stage 2 analysis using 200 simulations failed to reproduce the patterns observed in the PSP across the 19 fixed initializations suggesting that the observed PSP performance was not robust. Note that the 60–69% performance decile was separated into two patterns (7a and 7b) for a more fine-grained evaluation.

Figure 5 shows that the model performed poorly on the II categories. Approximately 60% of the parameter space performed at between 40 and 59% in the test phase, suggesting that, for more than half of the volume, the model produced no measurable learning. About 39% of the parameter space volume showed between 60 and 64% test accuracy and slightly more than 1% of the volume was above 65% test accuracy. The robustness analysis, described in Figure 7, shows that Figure 5 actually overestimates the performance of Model 1 (1FB-HS). While the test performance of the model clearly produces the expected output when averaged across the 19 fixed initializations (dark gray bars), every set of parameters (i.e., for each discovered data pattern) performs no better than 52% in the test phase when averaged across 200 random initializations (light gray bars). Because of the model's II categorization learning failure, the hybrid categories were not explored.

### Model 2: single source of feedback, soft-switch

Model 2 (1FB-SS) receives only one source of feedback [as in Model 1 (1FB-HS)], and uses soft switching [either system can potentially control the overall system response on every trial [as in Model 0 (2FB-SS)].

#### Information integration categories

The partitioning for Model 2 (1FB-SS) was comparatively diverse, suggesting that this model is capable of a wider range of behaviors. A total of 6 data patterns were discovered for the II categories (Figure 6, fourth column). The percentage of volume associated with each data region is also shown in Table 5. Although the model is capable of responding with accuracy above 90%, this behavior occurs only for a very small proportion of the parameter space. In fact, accuracy in the test phase was below 70% for approximately 80% of the parameter space. Overall, each data pattern appeared relatively robust across the 200 simulations. Only data patterns 3 and 4 performed worse (10 and 6%, respectively) on the 200 simulations than on the 19 fixed initializations. Hence, the performance of this model seems to be reliable.

**Table 5:** **Percentage of parameter space volume for each observed data pattern (or test phase accuracy ranges) for Model 2 (1FB-SS)**.

**Category**	**Test phase accuracy range (data patterns)**					
	**40–49%**	**50–59%**	**60–69%**	**70–79%**	**80–89%**	**≥90%**
II	0.002	17.075	61.113	20.718	0.949	0.143
Hybrid	0.032	54.875	28.374	13.036	3.556	0.127

Although only a small proportion (approximately 1%) of the overall parameter space was associated with test performance at or above 80% after training, the results suggest that for a restricted range of parameters, this model can successfully learn II categories with only a single source of feedback.

#### Hybrid categories

The performance of Model 2 (1FB-SS) on the hybrid categories was similar to its performance on the II categories. The same data patterns were observed, and the parameter space volumes associated with each of these patterns were similar (compare the 4th and 5th columns of Figure 5). When tested across the 200 random initializations, the model's performance was robust.

#### Soft switching drives learning in the procedural system

The ability of Model 2 (1FB-SS) to learn at all is in stark contrast to Model 1. The only difference between those models is the switching mechanism—Model 1 uses hard switching, whereas Model 2 uses soft switching. These results suggest that the switching mechanism is allowing the procedural system to learn in Model 2 (1FB-SS). For example, when the model switches to the procedural system, the procedural system receives veridical feedback, which should facilitate procedural learning. Note that this hypothesis predicts that model accuracy should increase with the proportion of trials controlled by the procedural system. To test this prediction, we computed the correlations between accuracy and the number of procedural system responses for Model 2 (1FB-SS) separately on the II and hybrid categories, both for the 19 training initializations and the 200 random initializations. All four of these correlations were *r* ≥ 0.97 (all *p* < 0.001).

Given that the procedural system only learns when it is parameterized so that it generates the majority (nearly 90%) of responses, a follow-up test was conducted to evaluate whether the procedural system would take over control of responding with simple RB categories (Figure 1, bottom panel). The RB categories were created by rotating the II categories (Figure 3) counter-clockwise so that the optimal bound (solid black line) is vertical.

The model was run for 200 simulations on these RB categories using the parameters that produced the best II learning (i.e., pattern 6). In these simulations, the RB performance was hard-coded so that the explicit system either performed at 90, 95, 99, or 100% accuracy. These simulations revealed that the average proportion of trials where the procedural system controlled responding was 0.84, 0.76, 0.41, and 0.00, respectively. This is fundamentally problematic because the explicit system should overwhelmingly control responding in RB tasks, especially when it is performing at such high accuracy levels.

## Discussion—simulation set 1

The results of the PSP analyses reveal the strength and limitations imposed by the feedback signal. First, when each system receives independent feedback (Model 0, 2FB-SS), the procedural system of COVIS readily learns to respond accurately in both II and hybrid categories. This is problematic because the model performs substantially better than humans in the hybrid task. Soft switching allows the model to pass control to the procedural system, which easily learns the hybrid categories. It is important to recall that Model 0 (2FB-SS) was designed deliberately to maximize learning. For this reason, our results represent a best-case scenario for this model.

In contrast, procedural learning was seriously compromised when both systems received the same feedback signal. Although the PSP for Model 1 hinted at a small amount of learning in the procedural system, that learning depended critically on the exact ordering of the stimuli during training, because during test the apparent learning disappeared. Note that Model 1 failed to learn even though the explicit system received correct feedback more than 75% of the time. This was because the feedback was independent of any activation within the procedural system, and thus it had the same effect as if the feedback was random.

Of course, the procedural system of Model 1 would learn *after* the hard switch occurs. But our results show clearly that the procedural system learns nothing until it controls responding in the task. Thus, in the II task simulated here, Model 1 predicts that accuracy should drop from around 75% correct to chance on the trial of the hard switch. As noted earlier, we know of no II studies that have reported such a mid-session drop in accuracy.

Finally, the results from Model 2 (1FB-SS) show that with soft switching, the procedural system of COVIS can learn provided that it controls responding for the majority of trials in the experiment. In other words, when the procedural system is allowed to generate the overall response for a large proportion of the training trials, it successfully learns to respond to the categories. That this occurs is unsurprising and essentially suggests that, within a narrow range of parameterizations, the model learns with one source of feedback in a serial fashion.

The empirical evidence (e.g., Ashby and Crossley, 2010) suggests that soft switching is not the dominant method via which humans resolve competition between declarative and procedural memory systems. However, other evidence (Erickson, 2008) suggests that a minority of humans seem to be able to switch trial-by-trial if given enough cues, so it may be the case that under some conditions, soft switching could occur naturally during the course of learning. When the task demands are clear, people may successfully adopt different strategies and flexibly shift among them. This is the idea behind knowledge partitioning, where participants learn to apply different strategies to different stimuli within one task (Lewandowsky and Kirsner, 2000; Yang and Lewandowsky, 2004).

Regardless of the plausibility of soft switching, the greatest problem with Model 2 (1FB-SS) is that it only predicts learning in the procedural system when the procedural system dominates responding in the task, regardless of whether that task is RB or II. This is problematic because the model predicts that even one-dimensional RB tasks will frequently be learned procedurally—a prediction that is strongly contradicted by the literature (e.g., Waldron and Ashby, 2001).

The results of these simulations largely suggest that with a single feedback source, simply modifying the system switching mechanism is not sufficient to account for human learning data. Regardless of the switching mechanism, the results show that learning in the procedural system is possible only if the procedural system controls responding throughout the majority of training.

## Additional COVIS modifications—the bootstrapping hypothesis

All of the models so far investigated predict no procedural learning while the explicit system controls responding. If we take for granted that the procedural system learns even while it does not control responding, then it appears that another modification to the architecture of COVIS may be necessary. One possibility is that the explicit system somehow trains or bootstraps the procedural system while it controls responding.

Although speculative, this could occur if the procedural system is somehow informed of the response the explicit system generates. As discussed above, the problem with the single feedback models so far investigated is that when the explicit system controls responding, the feedback it elicits is independent of activity within the procedural system. In other words, any change in cortical-striatal weights is as likely to be rewarded as any other change. If the procedural system is somehow fed information about the explicit system response, however, then this independence could disappear, which might allow the procedural system to learn from the explicit system.

### Implementing bootstrapping in COVIS

In order to implement bootstrapping in COVIS, a simple modification was made. Recall from earlier that, on every trial, the explicit system generates a discriminant value, *h*_*E*_(*n*), that quantifies the overall output of the explicit system's response. Also recall that, on every trial, each striatal unit produces an overall activation value that is driven by the stimulus. For example, activation in striatal unit J on trial *n* is denoted by *S_J_*(*n*) (i.e., see Equation 3). Using these values, the following modification was made to reflect the hypothesis that the procedural system is privy to the response generated by the explicit system whenever the explicit system controls responding. If the explicit system controls the overall response and emits a response corresponding to category *J* on trial *n*, set

(12) $$S_{J}(n)=S_{J}(n)+|h_{E}(n)|$$

and make no changes to *S*_*K*_(*n*), for all *K* ≠ *J*.

This modification makes no changes to the parameters of the model; it only makes a new assumption about the flow of information when the explicit system is generating the overall system response. Specifically, it assumes that information about the explicit system's response is fed back into the procedural system at the level of the striatum and only to the striatal unit matching the response of the explicit system. Ramping up the activity of the striatal unit corresponding to the response generated by the explicit system translates into larger changes in the weights associated with that striatal unit (see the reinforcement learning equation, Equation 4). The timing of this ramping-up of activity only needs to occur before feedback is given to the model, and because the explicit system discriminant value *h*_*E*_(*n*) is related to the explicit system decision, one can further assume that this information transfer occurs in tandem with the explicit system decision.

One attractive property of this modification is that it does not necessarily override the output of the procedural system. For example, suppose that the procedural system has been partially trained in an II categorization task, so its weights have been modified to respond accurately. The explicit system may make an incorrect “A” response to a stimulus, but because the procedural system has been trained, it is suggesting a correct “B” response with high confidence. The explicit system discriminant value *h*_*E*_(*n*) would be added to the striatal unit response *S_A_*(*n*), but *S_B_*(*n*) could still be larger and thus, the procedural system's output would not be washed out by the explicit system.

Early in training when the procedural system has not yet learned anything, both *S_A_*(*n*) and *S_B_*(*n*) will be approximately equal, so the explicit system's output will nudge learning in the procedural system in the direction of the explicit system's response when the weights are updated. The end result should be procedural system weights that are updated to reflect the response strategy of the explicit system as long as the explicit system controls responding.

## Simulation set 2—bootstrapped COVIS architectures

This section explores two COVIS architectures (Models 1 and 2) with bootstrapping implemented. The general methodology and presentation of results will follow those already described and presented. Bootstrapping Model 0 (2FB-SS) is unnecessary as it receives independent feedback in each system.

### Model 1: single source of feedback, hard switch, bootstrapped

This model is exactly as described above with the additional bootstrapping modification. It will be referred to as Model 1 (1FB-HS-B) because it receives only a single source of feedback (1FB), assumes hard switching, and is bootstrapped (B) by the explicit system.

### Model 2: single source of feedback, soft switch, bootstrapped

Model 2 (1FB-SS-B) is exactly as described above with the additional bootstrapping modification.

## Method—simulation set 2

The methods of assessment for both models were identical to those described for simulation set 1.

## Results—simulation set 2

### Model 1 (1FB-HS-B)

#### Information integration categories

The PSP on Model 1 (1FB-HS-B) was definitive: only one data pattern was discovered, corresponding to test phase accuracies greater than 90%. This suggests that across all parameter values (at least for the parameters defining the parameter space) the model learns II categories handily. The robustness analysis showed that the performance of the model across the 200 random simulations were essentially identical as across the 19 training simulations. Thus, the model is highly robust. Recall, however, that the suboptimal explicit strategy only performs at about 78% correct in II categories. How, then can the procedural system outperform the suboptimal strategy it was trained on?

**Figure 8:**
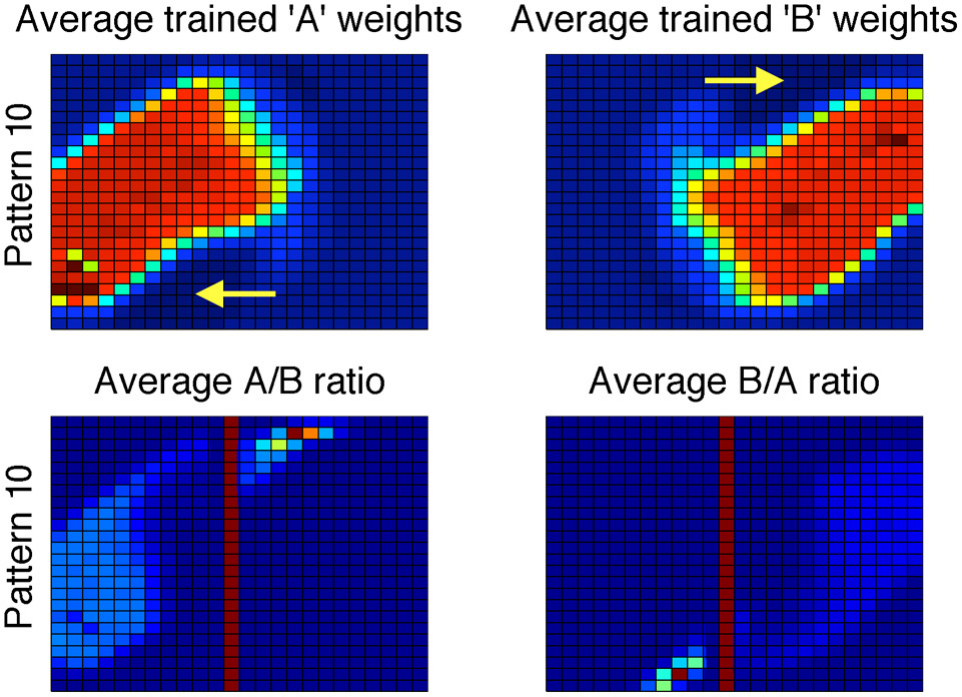
**Top:** Striatal weights in the procedural system of Model 1 (1FB-HS-B) trained on II categories, averaged across 200 simulations. Blue (cool colors) represent small weights whereas red (warm colors) represent large weights. Note the qualitative difference between these weights and those in Figure 6. **Bottom:** Ratio of procedural system weights. Solid vertical line approximately corresponds to the explicit system rule-based bound. Note that large values in the ratio correspond to regions where the weights in the denominator are driven toward zero (darkest blue regions in the top row indicated by yellow arrows).

A careful evaluation of the procedural system weights provides insight. The average trained procedural system weights across 200 simulations appear different than in previous models; the weights now show a residual trace of the vertical bound used by the explicit system (Figure 8, top). The effects of the explicit system training are most easily seen in the bottom of Figure 8, which shows a ratio of the A and B weights (i.e., A/B and B/A). Here, the solid red vertical line approximately corresponds to the boundary used by the explicit system. Note the small regions to the right and left of the bound in the A/B and B/A ratios, respectively. These are the regions where the procedural system weights are driven to zero due to the incorrect responses being made by the explicit system (top row, yellow arrows).

In other words, the procedural system not only learns to make the responses that the explicit system gets correct, but it also learns to avoid responses made by the explicit system that were incorrect. This analysis resolves the apparent paradox of how the procedural system was able to outperform the system that trained it.

#### Hybrid categories

The partitioning for Model 1 (1FB-HS-B) on the hybrid categories was similar to the II partitioning. The PSP algorithm discovered two data patterns corresponding to 80–89% test phase accuracy and greater than 90% test phase accuracy. The volume estimate was nearly 100% for the pattern corresponding to >90% test accuracy. The model's performance was also very robust across 200 simulations.

**Figure 9:**
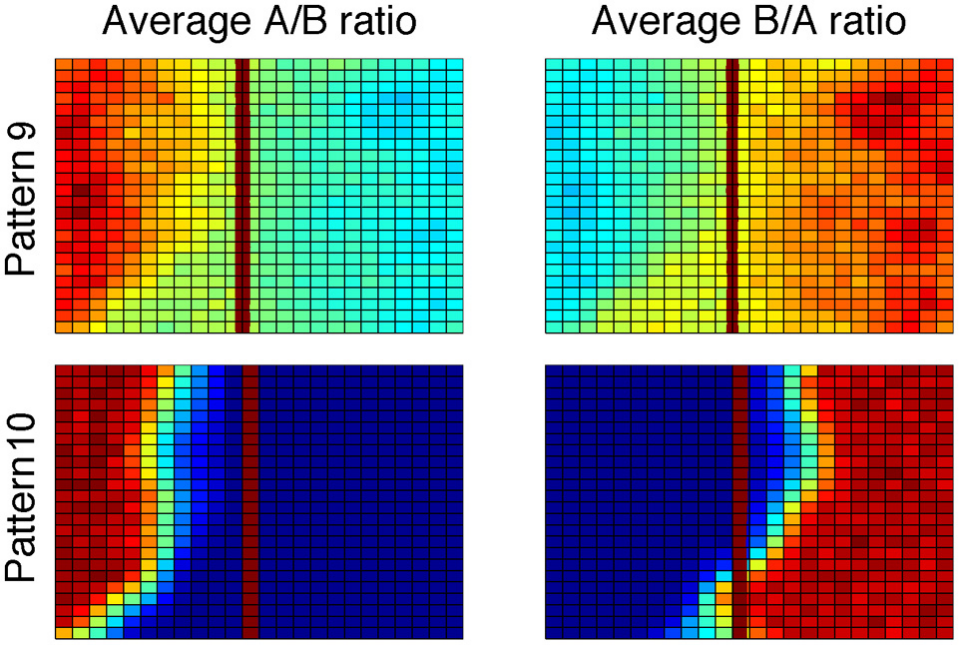
**Ratio of procedural system weights in Model 1 (1FB-HS-B) trained with hybrid categories**. Solid vertical line approximately corresponds to the explicit system rule-based bound.

As with the II categories, the weights learned by the procedural system show a residual trace of the explicit system training. This is most easily seen in Figure 9, which shows a ratio of the A and B weights. Note that, as with the II categories, the procedural system weights for the striatal unit where incorrect responses are made appear to be driven toward zero in the region where the explicit system responds incorrectly. Specifically, notice in the bottom right panel that there are some large positive values to the left of the explicit system's approximated bound. These large positive values in the ratio are due to the A-weights being very close to zero in those regions. Again, the procedural system learns what response not to make in this region.

### Model 2 (1FB-SS-B)

#### Information integration categories

The PSP analysis found only two data patterns corresponding to 80–89% and >90% test phase accuracy. Again, the volume of parameter space was nearly 100% for the >90% data pattern, and again, the model's performance was very stable across 200 simulations using random stimulus orderings and weight initializations. The pattern of weights learned by Model 2 (1FB-SS-B) were functionally identical to the weights learned by Model 1 (1FB-HS-B).

The only noteworthy difference between the models is that in Model 2 (1FB-SS-B), the procedural system is sometimes allowed to generate the overall system response. Recall that, in Model 2 (1FB-SS), the procedural system could learn, but only when the procedural system dominated responding (nearly 90% of all responses). This, however, was not a learning requirement in Model 2 (1FB-SS-B). Across 200 simulations, for pattern 9 (80–89% accuracy in the test phase), the procedural system only generated 7% of the overall responses on average; for pattern 2 (>90% test phase accuracy), the procedural system generated about 40% of the overall responses. Thus, procedural system learning in Model 2 (1FB-SS-B) is not critically dependent on the procedural system taking over the task.

#### Hybrid categories

Again, the PSP analysis only found two data patterns for Model 2 (1FB-SS-B) in the hybrid categories and nearly 100% of the volume of the parameter space was assigned to the pattern corresponding to >90% test phase accuracy. The performance of the model was also very robust across 200 simulations. The learned weights to those learned by Model 1 (1FB-HS-B).

Finally, as with the II categories, the success of Model 2 (1FB-SS-B) no longer hinged critically on the procedural system dominating the training phase. Across 200 simulations, the procedural system of Model 2 (1FB-SS-B) never accounted for more than 7% of the overall responses, on average (i.e., no parameterization caused the procedural system to dominate responding as before).

## Discussion—simulation set 2

With the addition of bootstrapping, there was a striking difference in the model's performance using only a single source of feedback. Basically, the bootstrapping modification allowed the feedback elicited by the responses of the explicit system to become useful to the procedural system. Model 1 failed to learn without bootstrapping whereas, with bootstrapping, it learned the categories well, even without ever generating a response. Model 2's performance previously depended on soft switching, which allowed the procedural system to dominate responding, but with the bootstrapping mechanism implemented, the model no longer required control.

Notably, the bootstrapping models learn both to respond and to not respond in certain regions of stimulus space, effectively allowing the procedural system to learn the categories better than the explicit system even though the procedural system only gets feedback based on the suboptimal explicit system strategy. This is a unique and strong prediction, which should be interpreted with some caution—recall that the models were biased toward learning very well, so it may be that, with a full explicit system and noise, the procedural system might not show such a dramatic performance improvement. This would be more in line with observed learning in human studies (i.e., learning curves generally have no large discontinuities from sudden drops or jumps in accuracy). However, even with noise, the weights in the procedural system should change in the same general fashion, so it seems that, with bootstrapping, the procedural system is capable of learning a little better than the explicit system, and once it is allowed to take over, refine its strategy.

Finally, it is interesting that in the soft switching Model 2 (1FB-SS-B), the procedural system was able to make as many as 40% of the overall responses with II categories, but not more than 7% with hybrid categories. This result is in line with the observation in Ashby and Crossley (2010) that humans tend to persist with suboptimal RB strategies with hybrid categories. Overall, learning in both models presented here is nearly identical. The current simulations, therefore, do not support a hard switching mechanism over a soft switching mechanism. With the assumption of bootstrapping, these simulations only confirm that the procedural system can learn with one source of feedback, regardless of the switching mechanism.

## General discussion

The simulations show clearly that the procedural system can learn with one source of feedback as long as the response generated by the explicit system is communicated back to the procedural system (i.e., via bootstrapping). Specifically, the model assumes that this information is passed back to the procedural system at the level of the striatum. COVIS is a model constrained by neurobiology, so although the simulations reported above verify that bootstrapping is a plausible computational mechanism that allows the procedural system to learn during explicit system control, an ideal model would identify a neurobiologically plausible explanation for how bootstrapping could work in a human brain.

There are a number of possible specific pathways via which the procedural system could receive an efferent copy of the explicit system motor response. In general, the challenge for all these accounts is that this efferent motor signal must project to the same striatal targets in the procedural system that receive the relevant visual input. Unfortunately, current neuroanatomy is not precise enough to draw any strong conclusions. Thus, the possibilities considered in this section must all be considered speculative. Hopefully, future research will clarify this issue.

The organizing scheme of the basal ganglia is characterized by parallel cortical-striatal-cortical projection loops (Alexander et al., 1986; Parent and Hazrati, 1995; DeLong and Wichmann, 2007). For this reason, one obvious hypothesis is that an efferent copy of the explicit system response is passed back to the striatum via direct cortical-striatal projections from premotor or motor areas of cortex to the striatal regions responsible for procedural learning. Within premotor areas, one intriguing possibility is that the signal originates in ventral premotor cortex (PMv), which is a likely candidate for the first motor-target of the explicit system.

Tracing and direct stimulation studies have found that premotor and motor regions project to distinct regions of the striatum (Takada et al., 1998; Nambu et al., 2002). Specifically, neurons from primary motor cortex (M1) send projections to medial aspects of the putamen, and neurons in premotor cortex project to dorsolateral regions of the putamen. Further evidence suggests connectivity patterns are both segregated and overlapping (Draganski et al., 2008), and also in accordance to the same somatotopic organization in cortex (Jones et al., 1977; Flaherty and Graybiel, 1993; Takada et al., 1998), which together suggest that the projections may indeed terminate in the general regions of the striatum responsible for executing the motor responses of the procedural system.

Recent studies have found different kinds of projections from M1 cortical layer V into the striatum. For example, Parent and Parent (2006) found not only direct projections from M1 into the striatum, but also indirect projections via long-range pyramidal tract neurons that form en passant synapses across wide regions. It is believed that these different projections actually stem from two different classes of layer V pyramidal neurons (Molnár and Cheung, 2006): those that send projections within the cortex and basal ganglia (IT-type) and those that send projections to deeper structures, brain stem, and spinal cord (PT-type; Reiner et al., 2003, 2010).

Another well-known organizing principle within the basal ganglia is the direct- and indirect-pathways (Albin et al., 1989; Gerfen, 1992; Pollack, 2001). Both pathways receive excitatory cortical input, but the direct pathway has the effect of increasing striatal output whereas the indirect pathway has the opposite effect. Some evidence now suggests that direct pathway striatal neurons receive input predominantly from IT-type neurons, whereas the indirect pathway neurons receive input largely from PT-type neurons (Reiner et al., 2003, 2010; Lei et al., 2004), and that short projecting IT-type neurons convey a different motor signal to the striatum than the motor signal conveyed to the spinal cord via long projecting neurons (Bauswein et al., 1989; Turner and DeLong, 2000). In contrast, the long projecting PT-type neurons may communicate to the striatum an efferent copy of the motor signals being sent to the spinal cord (Parent and Parent, 2006). Reiner et al. (2010) thus proposed a theory that these inputs ultimately lead to different kinds of motor modulation within the striatum. Specifically, they suggested that IT-type neuronal projections to the striatum facilitate planned motor actions along the direct pathway and that PT-type projections stymie conflicting motor actions along the indirect pathway.

This hypothesis suggests that projections to the indirect pathway might teach the procedural system what responses not to make early on during explicit system control by driving weights toward zero in regions where the suboptimal explicit strategy yields incorrect responses. Similarly, projections to the direct pathway could teach the procedural system when there is agreement between the explicit system's strategy and the categories by increasing weights. Broadly speaking, the differential effects of these cortical-striatal projections would translate to increasing one striatal response and decreasing another, which has the overall effect of increasing the output of the elicited motor response relative to the not-elicited response. Although there is no indirect pathway in COVIS, this is computationally the effect achieved by adding the output of the explicit system to the procedural system. Furthermore, it would be straightforward to implement COVIS with the addition of the indirect pathway. Even if this dichotomy between projections to the striatum is incorrect, the existence of projections from cortical motor regions to the striatal nuclei hypothesized to mediate procedural learning is well-established, and thus a plausible pipeline through which explicit system responses are communicated to the procedural system.

A very different alternative possibility is that the explicit system executes its motor response by activating the striatum, in which case the information about its response might automatically be communicated to the striatum. In other words, this hypothesis predicts that the striatum is involved in explicitly produced, volitional movements, either at the level of generation or execution of motor movements. Striatal involvement in volitional movement is corroborated by PET (Roland et al., 1982; Jueptner and Weiller, 1998), and fMRI (Cunnington et al., 2002) experiments. Neurophysiological studies in non-human primates provide more direct evidence (e.g., Romo et al., 1992; Schultz and Romo, 1992). In those experiments, striatal neurons responded to self-initiated movement, either leading up to the movement (suggesting involvement in generating the motor action), or with the movement (suggesting involvement in executing the motor action).

Although the exact computational mechanism is currently unknown, the available neurobiological evidence supports the possibility that the procedural system of COVIS can be bootstrapped by the explicit system before it takes over responding in a perceptual category-learning task. This bootstrapping could be from cortical-striatal projections from premotor or motor regions into the striatum, or possibly by the explicit system's control of motor responses through basal ganglia-mediated loops.

### Conflict of interest statement

The authors declare that the research was conducted in the absence of any commercial or financial relationships that could be construed as a potential conflict of interest.
